# Fostering belonging and advancing engagement: The impact of the Employee and Community Engagement Committee at the University of California, Los Angeles Immunogenetics Center

**DOI:** 10.1016/j.acpath.2026.100301

**Published:** 2026-09-23

**Authors:** Krystal Kendall, Belen Alfaro, Andrea Alvarez, Bernard Baylosis, Arlene Locke, Nora Garcia, Abiha Hasnain, Elaine F. Reed, Rebecca A. Sosa

**Affiliations:** a Department of Pathology and Laboratory Medicine, David Geffen School of Medicine at UCLA, Los Angeles, CA, USA; b UCLA Immunogenetics Center, Los Angeles, CA, USA

**Keywords:** Engagement, Inclusion, Professional development, Workplace culture

## Abstract

The University of California, Los Angeles Immunogenetics Center established an Employee and Community Engagement Committee in 2022 to cultivate an inclusive workplace culture, enhance employee well-being, and foster meaningful connections with local and broader communities. This article presents an overview of the Employee and Community Engagement Committee's organizational framework, representative activities, and measurable outcomes from 2022 through 2025. The multidisciplinary committee, composed of technologists, administrative staff, faculty, and trainees, meets regularly and reports directly to University of California, Los Angeles Immunogenetics Center leadership, ensuring alignment with departmental and institutional priorities. The Employee and Community Engagement Committee has implemented a wide range of initiatives designed to improve workplace recognition, promote wellness, and extend outreach. Examples include guided meditation and fitness challenges, cultural heritage celebrations, staff appreciation events, and creative recognition efforts such as peer-to-peer acknowledgments. Community service projects have involved partnerships with organizations including Project Angel Food, the University of California, Los Angeles Volunteer Center, and the American Heart Association, alongside longstanding traditions such as the Annual Pumpkin Carving Contest supporting the University of California, Los Angeles Intervention Program. Professional development activities encouraged participation in institutional learning opportunities and conferences, raising the profile of the laboratory within the immunogenetics field. Social media engagement further amplified these efforts, enhancing recruitment and visibility. Evaluation of outcomes demonstrated measurable improvements in employee satisfaction, increased participation in voluntary events, and reduced staff turnover. Externally, the Employee and Community Engagement Committee strengthened partnerships across University of California, Los Angeles and Los Angeles, expanded participation in outreach activities, and enhanced visibility of University of California, Los Angeles Immunogenetics Center's mission. Collectively, this model demonstrates how engagement committees can institutionalize belonging and social responsibility, offering a framework that may be adapted by other academic pathology departments.

## Introduction

Employee engagement and community outreach are critical components of a thriving academic healthcare environment.^1^^,^^2^ In laboratory medicine and pathology, where much of the workforce operates behind the scenes, fostering visibility, connection, and professional fulfillment presents unique challenges. High workload demands, limited direct patient interaction, and siloed workflows may contribute to disengagement and burnout if not proactively addressed.

At the University of California, Los Angeles (UCLA) Immunogenetics Center (UIC), a high-volume laboratory specializing in histocompatibility and immunogenetics testing, sustained efforts to support employee wellness and community engagement have become essential to our mission. In response to both internal feedback and broader institutional priorities, the Employee and Community Engagement Committee (ECEC) was formed to build a culture of belonging and connect our work to the communities we serve.^3^^,^^4^

Although employee engagement and community outreach are often conceptualized as distinct institutional priorities, they may be synergistic within academic healthcare environments. Employee engagement initiatives primarily aim to improve workplace culture, well-being, and retention, whereas community outreach efforts focus on external partnerships, service, and public engagement.^1^^,^^2^^,^^5^, ^6^, ^7^ However, emerging frameworks in academic medicine and organizational culture suggest that linking internal engagement with external, mission-driven activities may reinforce a shared sense of purpose, enhance job satisfaction, and strengthen institutional identity.

At the UIC, these domains were intentionally integrated within a single committee structure based on the hypothesis that connecting staff engagement with community-facing initiatives would amplify both internal morale and external impact. This combined model allows employees to see the broader significance of their work while simultaneously fostering collaboration, inclusivity, and institutional visibility. To our knowledge, there is limited published literature describing this integrated approach within academic pathology or laboratory medicine, highlighting the need for structured evaluation of such models.

Although many institutions recognize the importance of workplace culture and community engagement, there remains limited published literature describing structured, laboratory-based engagement programs and their measurable impact within academic pathology settings. Existing reports have largely focused on physician engagement or institutional initiatives, with fewer examples detailing multidisciplinary laboratory teams or sustained programmatic approaches.

Social media has also emerged as an important tool for academic pathology departments to enhance visibility, promote professional engagement, and disseminate institutional activities to broader audiences.^8^ When integrated with internal engagement efforts, these platforms may further amplify institutional culture, support recruitment, and extend community outreach beyond traditional boundaries.

In this study, we describe the development, structure, and implementation of the ECEC at the UIC and evaluate its impact on employee engagement, community outreach, and institutional visibility from 2022 through 2025. Our aim is to provide a practical and scalable framework that may be adapted by other academic pathology and laboratory medicine programs seeking to enhance workplace culture and community engagement.

## Materials and methods

This study represents a retrospective, descriptive evaluation of an institutional engagement program conducted at the UIC from 2022 through 2025. The objective was to characterize the structure, implementation, and outcomes of the ECEC using predefined engagement and participation metrics.

### Program design and organizational structure

The ECEC was established in 2022 as a multidisciplinary, voluntary body that includes clinical technologists, administrative staff, faculty, and trainees. Established to strengthen organizational culture within the UIC,^9^ the committee pursues several core objectives: enhancing internal communication and cohesion; supporting staff development and recognition; promoting health and wellness in the workplace; celebrating cultural and professional contributions; and encouraging community outreach and volunteerism.

The ECEC reports directly to the UIC leadership team and convenes for a half hour twice weekly to plan, implement, and evaluate initiatives. The ECEC convenes twice weekly for brief, 30-min sessions designed to accommodate staff across multiple shifts and operational areas. These meetings are structured to allow flexible participation, with members attending as needed based on ongoing initiatives. This approach enables inclusive engagement while maintaining efficiency, with additional coordination and final decision-making conducted asynchronously via email or institutional communication platforms when appropriate. Through this structure, the committee ensures that diverse perspectives are represented and that activities are aligned with both departmental priorities and institutional values. This structure facilitated timely planning and execution of initiatives while minimizing disruption to clinical operations.

For the purposes of evaluation, ECEC activities were organized into two primary domains: (1) employee engagement (including recognition, wellness, and professional development) and (2) community outreach and external engagement.

### Program components

***Employee recognition and engagement***. Fostering a culture of recognition and appreciation has been a central priority of the ECEC, with initiatives designed to acknowledge staff contributions and strengthen engagement across all levels of the Immunogenetics division of the Department of Pathology at UCLA.

Recognition efforts have included work anniversary announcements, routine “spotlights” on social media highlighting individual achievements, and in-person “shout-outs” during staff meetings. These were complemented by broader engagement activities such as employee appreciation days and themed events. Examples are summarized in Table 1.

**Table 1:** Representative employee engagement and recognition activities implemented by the UIC Employee and Community Engagement Committee (ECEC).

Activity type	Example activities
Wellness and recreation	Meditation sessions, chair massages, and interactive games to support employee well-being
Festive spirit weeks	Department-wide participation during holidays or other celebratory times
Life milestones	Celebrations of weddings, graduations, baby showers, and other significant personal achievements
Seasonal traditions	The annual pumpkin carving contest and additional holiday-themed activities, such as: • Distributing Valentine's cards with candy • Creating a gratitude wall with leaf cutouts at Thanksgiving • Crafting homemade ornaments for UIC's Christmas tree
Peer recognition initiatives	Creative approaches such as: • “Awesome citations,” which encouraged staff to write playful, handwritten notes acknowledging colleagues' efforts • Candy-gram distributions to thank teams across the laboratory for their contributions

UIC: University of California, Los Angeles (UCLA) Immunogenetics Center.

Together, these recognition and engagement initiatives were designed to facilitate staff engagement and promote community involvement across the division.^1^^,^^10^

***Professional development***. To encourage continuous growth and expertise within our department, we actively supported staff participation in institutional learning and development programs and professional conferences such as the American Society for Histocompatibility and Immunogenetics (ASHI) and the Annual Human Leukocyte Antigen (HLA) & Transplant Workshop sponsored by One Lambda/Thermo Fisher. These courses provided opportunities for employees to gain professional skill and develop their expertise. Employees were encouraged to apply to attend and present their work at relevant conferences, providing valuable opportunities for skill development, networking, and academic recognition. These experiences also helped raise the profile of our division within the immunogenetics community.

***Wellness and mental health programming***. As part of its commitment to fostering a healthy and supportive work environment, the ECEC organized a series of wellness and health-focused initiatives for staff. Programming included guided meditation and sound bath sessions, department-wide fitness challenges, on-site visits from UCLA's FitWell team for guided stretching exercises, and therapeutic massage sessions provided by licensed professionals.

***Community outreach and engagement***. Community outreach represents a central objective of the ECEC. The committee is dedicated to contributing meaningfully to both the broader UCLA and Los Angeles communities, while simultaneously fostering opportunities for staff to connect, celebrate, and support one another within the workplace.^5^, ^6^, ^7^ The following provides a representative overview of past outreach activities:

• *Service initiatives:* University of California, Los Angeles Immunogenetics Center staff have participated in food drives, local blood donation campaigns, and partnerships with organizations such as Project Angel Food and the UCLA Volunteer Center. A long-standing effort has been our annual participation in UCLA's event to assemble care packages and hygiene kits for individuals experiencing homelessness in the Los Angeles community.
• *Organ procurement organization (OPO) engagement:* Staff join the OPO's annual run/walk, providing opportunities to connect with organ recipients, donors, and donor families.
• *American Heart Association Heart and Stroke Walk (2024):* Our department joined this UCLA-wide initiative, adding a friendly competition with a neighboring laboratory to increase participation.
• *Science, Technology, Engineering and Mathematics (STEM) education outreach:* The annual “I Have a Dream in STEM” Conference was launched at UCLA in 2023. University of California, Los Angeles Immunogenetics Center participated with the department's Mini Pathologist's PlayLab, offering elementary and middle school students a hands-on introduction to laboratory medicine through interactive stations and mentorship. These activities were designed to allow students to explore how pathology and laboratory medicine contribute to patient care, while fostering early interest in STEM careers.^9^
• *Annual Pumpkin Carving Contest:* Established in 1994, this tradition engages UCLA staff and families while supporting the UCLA Intervention Program, which provides therapeutic and educational services for children and their families. Children in costumes enjoy trick-or-treating while viewing decorated pumpkins created by UIC staff.
• *Patient engagement:* The committee organized in-person talks from patient speakers, including guided tours of the laboratory and personal stories of transplantation, helping staff connect their daily work to patient outcomes.
• *Community and wellness projects:* Staff participated in the Semel Institute Gardening Project, contributed to a dog adoption event, and attended the annual departmental picnic, further strengthening community ties and work-life balance.

These efforts have contributed to a more connected and positive work environment by creating meaningful opportunities for staff to engage with one another and to give back to the wider community. Collectively, they demonstrate the committee's ongoing commitment to building a workplace that values appreciation, connection, and community involvement.

***Social media presence***. Social media platforms are increasingly recognized as valuable tools for professional engagement, recruitment, and dissemination of academic activities within pathology.^8^ The ECEC has implemented a multiplatform social media strategy across Instagram and LinkedIn to increase the visibility of histocompatibility and immunogenetics. The purposes of each platform are summarized in Table 2.

**Table 2:** Social media platforms and their primary roles in supporting engagement, communication, and outreach at the UIC.

Social media platform	Purpose
Instagram	Highlight departmental culture through event recaps, employee spotlights, and creative series, drawing consistent engagement from staff and external audiences
LinkedIn	Professional outreach such as but not limited to job postings in addition to spotlighting publications, conference participation, and achievements by our faculty and staff

UIC: University of California, Los Angeles (UCLA) Immunogenetics Center.

### Evaluation and metrics

Outcomes of ECEC initiatives were evaluated using both quantitative and qualitative measures. Metrics were analyzed within these domains to assess both internal (employee-focused) and external (community-facing) impact. Quantitative measures included participation data (e.g., number of event participants, contest entries, and ballots cast), social media analytics (e.g., engagement rate, impressions, and reach), and selected operational indicators such as staff participation trends over time. Social media data were obtained from platform-specific analytics tools (LinkedIn Analytics and Meta Business Suite for Instagram).

Qualitative assessment included informal feedback from staff and participants, as well as observational assessment of engagement across activities. Given the programmatic nature of this initiative, formal survey instruments were not systematically implemented during the study period.

This study was conducted as a quality improvement and program evaluation initiative and did not involve human subjects research as defined by institutional guidelines; therefore, formal Institutional Review Board approval was not required.

## Results

Outcomes were evaluated across two primary domains: employee engagement and community outreach, with social media serving as a cross-cutting tool supporting both areas. Evaluation metrics were selected to align with established frameworks for program assessment, including elements of the Kirkpatrick model.

### Employee engagement metrics

Participation in ECEC-supported activities increased over the study period (2022–2025), with 2022 representing the initial implementation phase and serving as a practical baseline for subsequent comparisons. The Annual Pumpkin Carving Contest, a recurring divisional event, demonstrated progressive growth following its reintroduction after the COVID-19 pandemic (2020–2021). Participation increased from 11 entries and 80 ballots cast in 2022 to more than 100 ballots in 2023, reaching a peak in 2024 with 27 individual entries, three group entries, and 285 ballots cast. In 2025, participation included 19 contestants and 227 ballots cast (Fig. 1, Fig. 2).

**Fig. 1:**
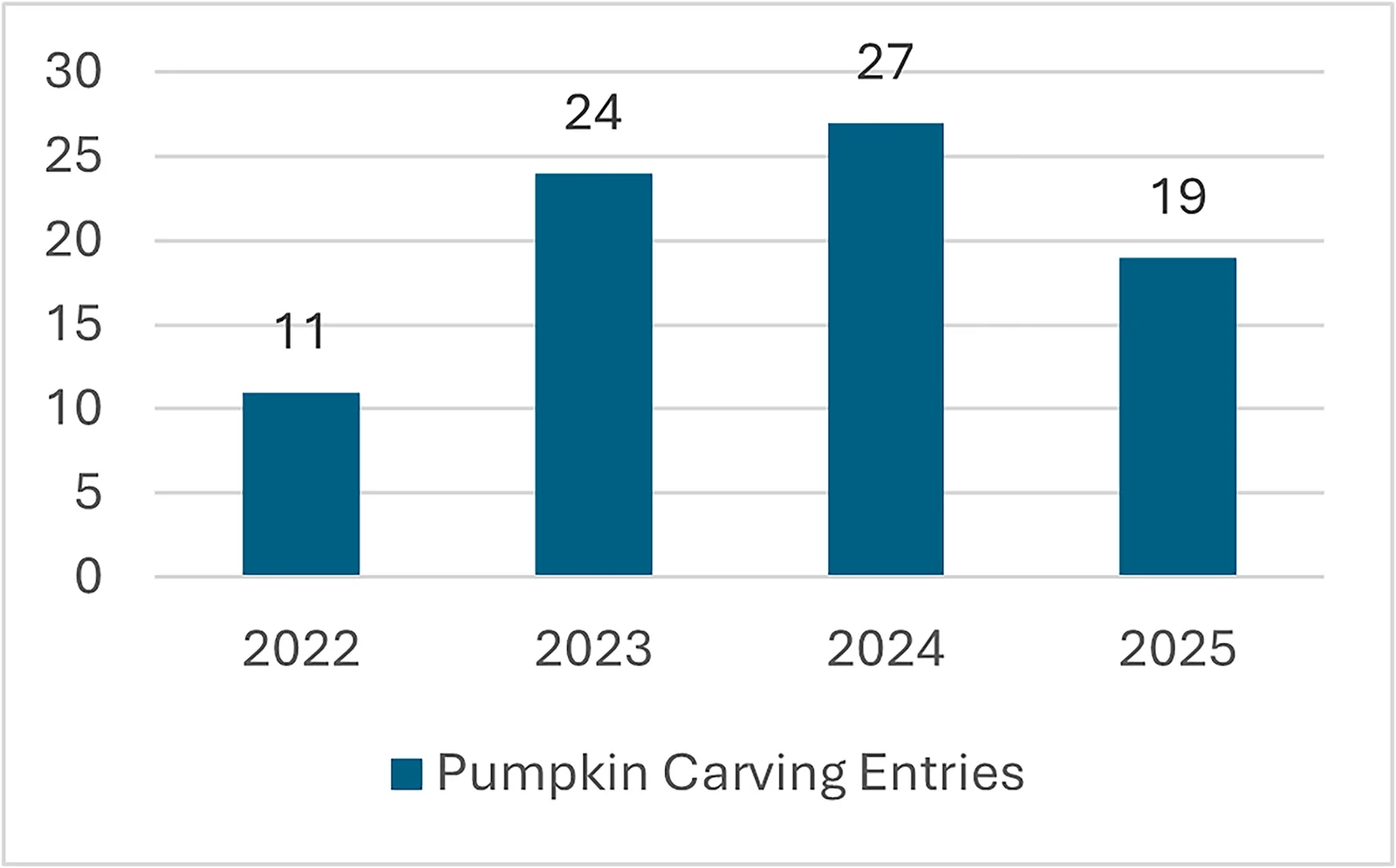
Annual Pumpkin Carving Contest entries at the UIC (2022–2025). The number of individual and group entries increased following the reintroduction of the event in 2022, demonstrating growing staff participation in engagement activities over time. UIC: University of California, Los Angeles (UCLA) Immunogenetics Center.

**Fig. 2:**
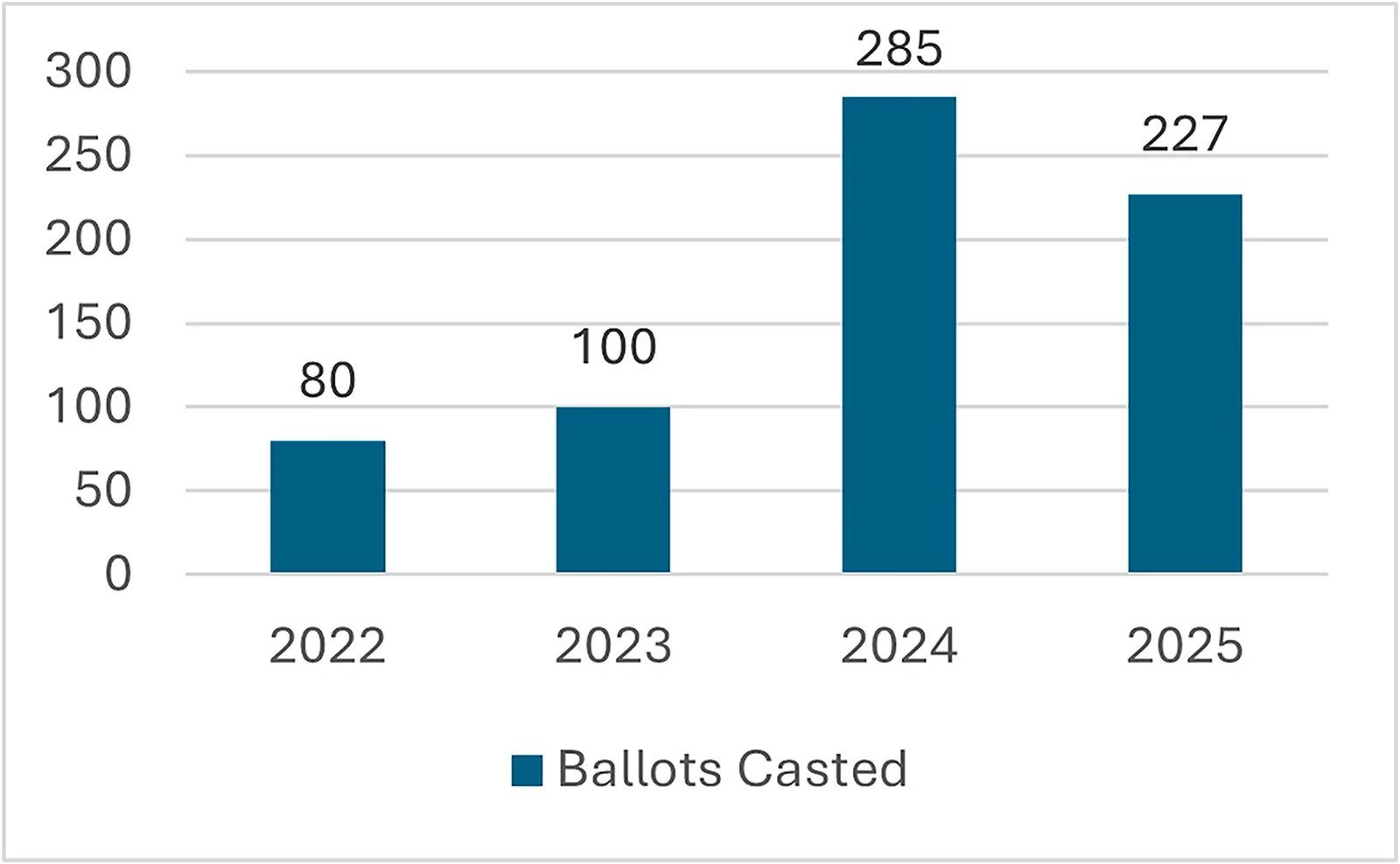
Voter participation in the Annual Pumpkin Carving Contest (2022–2025). The number of ballots cast increased substantially after 2022, reflecting increased staff engagement and participation in departmental activities.

Although ballot participation declined modestly in 2025 than 2024, levels remained substantially higher than the baseline in 2022. The increase coincided with expanded social media and promotional activities.

### Community outreach metrics

Employee and Community Engagement Committee-supported outreach initiatives expanded over time, with increased staff participation and external engagement. In the 2024 American Heart Association Heart and Stroke Walk, departmental participation reached 31 walkers compared to 28 walkers from a partner laboratory, contributing to a total of $5151 raised and exceeding the departmental fundraising goal.

Participation in additional outreach activities, including STEM education initiatives, service projects, and community-based events, also increased over the study period, reflecting broader staff involvement in external engagement efforts.

### Social media analytics

Social media activities supported both employee engagement (e.g., recognition and internal culture) and community outreach (e.g., external visibility and recruitment). Social media metrics demonstrated measurable growth in institutional visibility and engagement. On LinkedIn, content posted between September 2024 and January 2026 achieved an average engagement rate of 12.3%, with a total reach of 3225 unique users and growth to 322 followers.

Instagram analytics from Meta Business Suite documented 24,029 content views, 1354 profile visits, and 460 content interactions over the past year. The audience included users across multiple geographic regions, including North America, South America, Europe, Asia, and the Middle East.

Post categories were tracked to assess content strategy and audience engagement patterns. Content categories contributing to engagement are summarized in Fig. 3, which illustrates the distribution of posts across themes such as community outreach, employee recognition, professional development, and departmental activities.

**Fig. 3:**
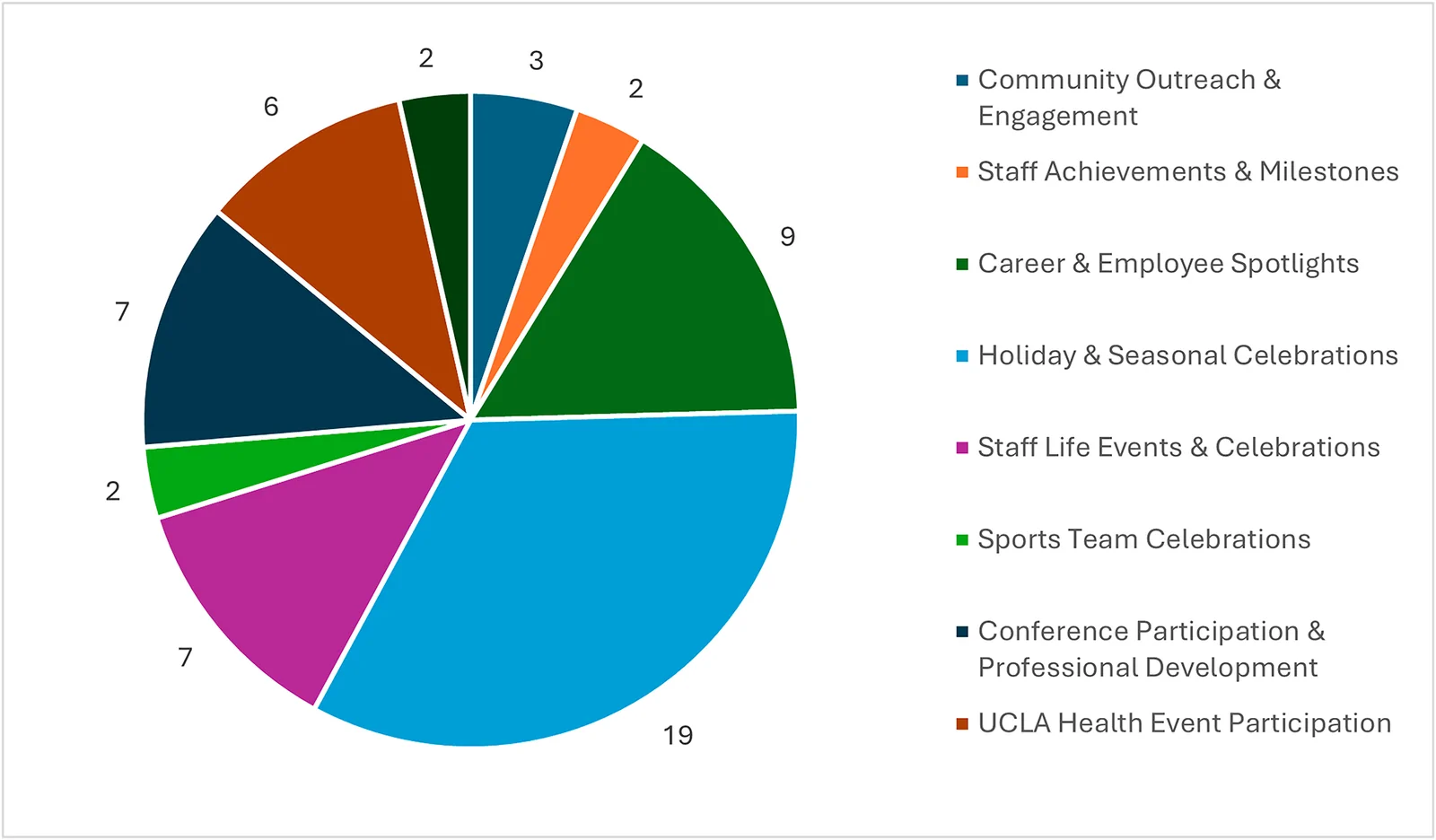
Distribution of Instagram post categories for the UIC. Posts were categorized into thematic groups, including community outreach and engagement, staff achievements and milestones, career and employee spotlights, holiday and seasonal celebrations, professional development, and departmental activities. The distribution reflects the content strategy used to promote engagement and highlight institutional culture. UIC: University of California, Los Angeles (UCLA) Immunogenetics Center.

Content engagement was highest on Instagram and LinkedIn, which were therefore prioritized as primary platforms for outreach and communication. Several job candidates reported during interviews that exposure to the department's social media presence positively influenced their perception of the workplace environment.

### Internal impact and qualitative observations

Employee and Community Engagement Committee initiatives were associated with increased staff participation in wellness and engagement activities, including fitness challenges and institutional wellness programming. These initiatives promoted participation in health-focused activities and increased awareness of available institutional resources, such as the Employee Assistance Program (EAP). The fitness challenges emphasized healthy eating and regular physical activity, encouraging broad participation across the division. Staff members tracked their individual progress throughout the initiative, which culminated in a recognition announcement honoring top participants based on their overall achievements. In collaboration with UCLA Staff Assembly, the ECEC also promoted awareness and utilization of EAP resources. Collectively, these efforts were associated with the promotion of healthy lifestyle habits, reduction of workplace stress, and reinforcement of a culture of care and well-being within our division and the department.^1^^,^^11^

Qualitative feedback from staff indicated improved cross-functional interaction, increased visibility of junior team members, and enhanced opportunities for participation across roles. Staff emphasized that the committee provided a visible and trusted platform for amplifying the voices of junior colleagues and individuals from groups historically underrepresented in academic medicine.^3^ Anecdotal observations also suggested that the ECEC contributed to reducing organizational silos and fostering a more inclusive workplace environment. These efforts were perceived by participants to foster a stronger sense of belonging while ensuring that innovative ideas and diverse perspectives were incorporated into departmental initiatives. Although formal quantitative tracking of professional development participation was not systematically implemented during the study period, increased attendance at conferences and institutional training programs was observed over time based on departmental records and anecdotal reporting. Formal institutional employee engagement survey data were not analyzed as part of this study due to limitations in data accessibility and the proprietary nature of externally administered surveys.

### Recruitment and institutional visibility

Several job candidates reported during interviews that exposure to the department's social media presence positively influenced their perception of the workplace environment. This observation suggests a potential role for social media engagement in recruitment and institutional branding.

## Discussion

These findings highlight the potential for structured engagement programs to serve as scalable models for improving workplace culture and institutional visibility within academic pathology. Across multiple domains, including event participation, community outreach, and social media engagement, the ECEC demonstrated measurable and sustained growth, supporting its role as an effective mechanism for fostering connection and engagement within a laboratory-based setting. Importantly, our findings support the hypothesis that integrating employee engagement with community outreach may create reinforcing effects, whereby internal culture strengthens external impact and vice versa.

The observed increase in participation metrics, particularly in recurring events such as the Annual Pumpkin Carving Contest, suggests that consistent programming and visibility can drive sustained engagement over time. Similarly, expansion of community outreach activities and increased fundraising participation reflect growing staff investment in initiatives that extend beyond the laboratory, reinforcing a sense of shared purpose and institutional identity. Social media analytics further support the impact of the ECEC, demonstrating measurable gains in reach, engagement, and audience diversity. These findings align with prior reports highlighting the value of social media in academic pathology for professional visibility, recruitment, and dissemination of institutional activities.^8^ In our experience, integrating social media with internal engagement efforts amplified these effects, extending the impact of local initiatives to a broader audience.

The impact of the ECEC can also be interpreted using the Kirkpatrick model for program evaluation, which provides a structured framework for assessing educational and organizational interventions across four levels: reaction, learning, behavior, and results.^12^ At the level of reaction, increased participation in engagement activities and positive qualitative feedback from staff suggest that initiatives were well received. At the level of learning, although formal pre- and post-assessment tools were not implemented, participation in professional development activities and increased awareness of institutional resources indicate potential gains in knowledge and engagement. Behavioral changes were reflected in increased staff participation (employee engagement), and expanded involvement in outreach activities (community engagement), and cross-functional collaboration, suggesting a shift in workplace engagement patterns. At the highest level, results were demonstrated through measurable increases in participation metrics, expanded community outreach, and enhanced institutional visibility through social media. However, direct measurement of downstream outcomes such as staff retention, patient care impact, or operational efficiency was beyond the scope of this study. Future work incorporating standardized engagement surveys and longitudinal outcome measures will be important to more fully evaluate these higher-level effects.

The success of the ECEC is grounded in strong leadership support, consistent communication, and responsiveness to staff needs. Importantly, the multidisciplinary composition of the committee, including technologists, administrative staff, faculty, and trainees, enabled broad representation and facilitated cross-functional collaboration. This structure may be particularly relevant for laboratory medicine environments, where hierarchical and operational silos can otherwise limit engagement.

A key limitation of this study is the absence of systematically collected preintervention baseline data for several domains, particularly professional development and learning outcomes. While certain activities, such as the Annual Pumpkin Carving Contest, provided clear year-over-year comparisons that function as proxy baseline-to-follow-up measures, other initiatives were implemented without formal quantitative tracking at baseline. As a result, observed increases in participation and engagement in these areas are supported primarily by longitudinal trends and qualitative observations rather than direct pre- and post-intervention comparisons. Future iterations of this program will incorporate structured baseline assessments and standardized tracking of participation in professional development and educational activities to enable more rigorous evaluation. To enhance the practical applicability of this model, key lessons learned and implementation strategies are summarized in Table 3. These insights may inform similar efforts in other academic pathology and laboratory medicine settings.

**Table 3:** Key lessons learned and implementation strategies for engagement programs in clinical laboratory settings.

Lesson learned	Practical strategy	Relevance to other institutions
Leadership support is critical for sustainability	Establish direct reporting structure to institutional leadership and align initiatives with departmental priorities	Ensures visibility, resource support, and long-term program viability
Multidisciplinary representation enhances engagement	Include technologists, administrative staff, faculty, and trainees in committee membership	Promotes inclusivity and ensures diverse perspectives are incorporated
Flexible meeting structures improve participation	Use brief (30-min), optional meetings with asynchronous follow-up via email or teams	Particularly important in shift-based clinical laboratory environments
Integration of engagement and outreach strengthens impact	Combine internal engagement initiatives with community-facing activities	Reinforces shared purpose and institutional identity
Social media amplifies engagement and recruitment	Use platforms such as Instagram and LinkedIn to highlight activities and achievements	Enhances visibility, recruitment, and external engagement
Consistent programming drives sustained participation	Maintain recurring events (e.g., annual traditions) and build on prior successes	Supports longitudinal engagement and culture building
Addressing scheduling challenges requires adaptability	Rotate leadership roles and allow flexible participation	Helps maintain momentum in busy clinical environments
Formal evaluation methods should be incorporated early	Implement baseline and longitudinal tracking of engagement metrics and surveys	Enables more rigorous assessment of program impact

Key challenges included coordinating activities across variable staff schedules and maintaining volunteer momentum. These were addressed through strategies such as rotating committee leadership roles and embedding engagement activities into departmental operations, thereby ensuring sustainability. The use of brief, flexible meeting structures supported broad participation across staff roles and schedules, which may be particularly important in clinical laboratory environments with shift-based workflows.

Additionally, although the study incorporates several quantifiable metrics, it does not yet include comprehensive pre- and post-intervention surveys or a full set of standardized outcome measures. Future work will focus on implementing validated assessment tools to more rigorously evaluate changes in employee engagement, participation, and retention. Although qualitative feedback and participation trends suggest improvements in workplace engagement and morale, this study did not incorporate standardized institutional survey data to quantitatively assess these outcomes. Institution-wide employee engagement surveys are administered separately and are subject to access and reporting limitations, restricting their use in this analysis. As a result, conclusions regarding changes in morale and workplace satisfaction are based on indirect measures, including participation metrics and qualitative observations. Future studies integrating validated engagement surveys and pre- and post-intervention assessments will be important to more rigorously evaluate these outcomes.

## Conclusion

The UIC's ECEC has become an important driver of institutional cohesion and community service. By fostering a supportive workplace culture and expanding engagement beyond the laboratory, the committee has strengthened both internal morale and external visibility. Importantly, this model demonstrates that structured engagement initiatives can be successfully implemented within high-volume clinical laboratories without compromising operational demands.

Our experience suggests that similar programs may be adapted across academic pathology and laboratory medicine settings, with flexibility to align with institutional priorities and available resources. As displayed in Fig. 4, this framework may serve as a practical model for other departments seeking to implement sustainable employee and community engagement initiatives.

**Fig. 4:**
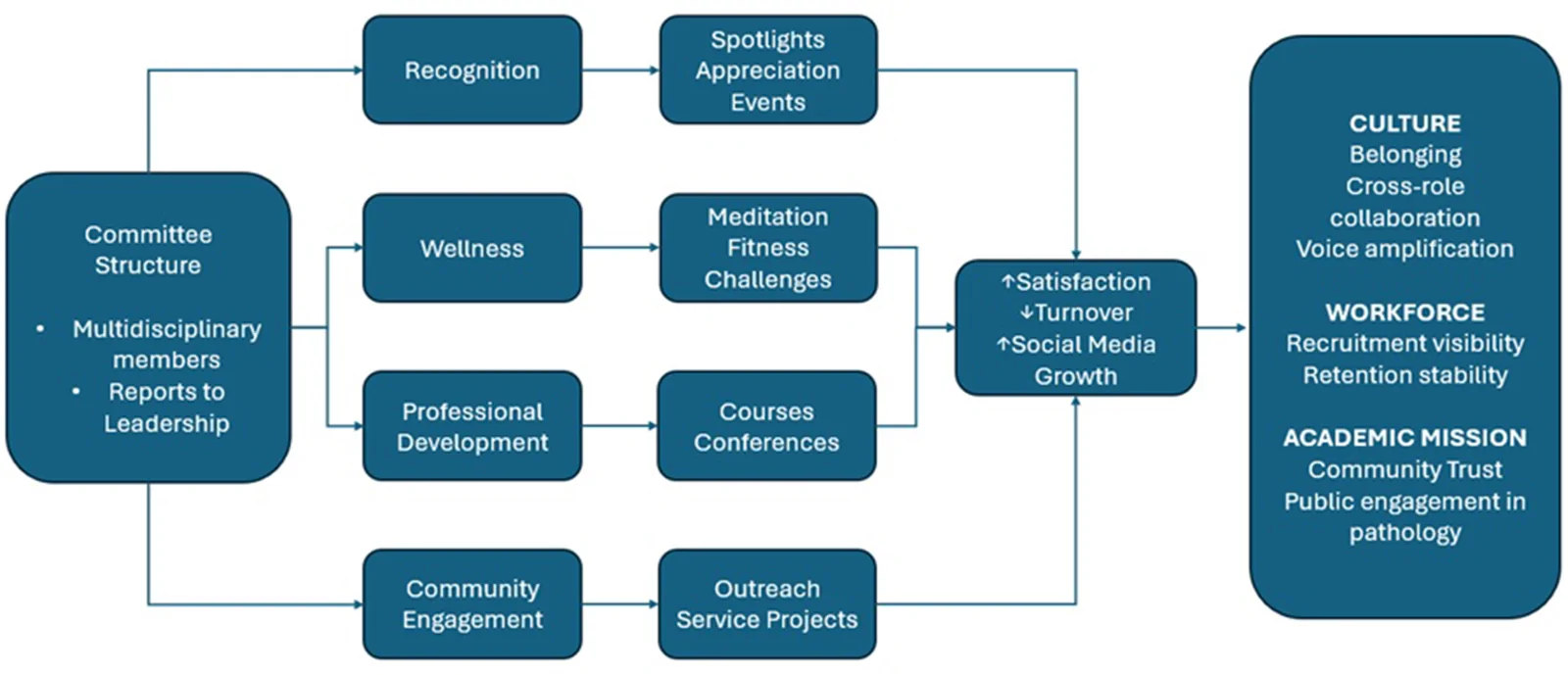
Framework and impact of the UIC employee and community engagement committee. A multidisciplinary governance structure enabled coordinated programming across four domains (recognition, wellness, professional development, and community engagement), leading to measurable improvements in employee satisfaction, participation, recruitment visibility, and institutional outreach. UIC: University of California, Los Angeles (UCLA) Immunogenetics Center.

## Declaration of competing interest

The authors declare that they have no known competing financial interests or personal relationships that could have appeared to influence the work reported in this paper.
